# Identification and validation of the potential therapeutic value of CASK in osteosarcoma: a computational analysis and *in vitro* experiments

**DOI:** 10.3389/fonc.2026.1693570

**Published:** 2026-03-16

**Authors:** Hongjuan Yang, Weiying Zhong, Danping Shen, Sihui Chen

**Affiliations:** Department of Orthopaedics, Jiaxing University Affiliated Hospital, The First Hospital of Jiaxing, Jiaxing, China

**Keywords:** CASK, immune infiltration, osteosarcoma, transcriptome, U2OS

## Abstract

**Introduction:**

Currently, identifying new therapeutic targets in clinical practice is crucial for improving the treatment of osteosarcoma. The abnormal expression of calcium/calmodulin-dependent serine protein kinase (CASK) plays an important role in the tumorigenesis of many cancers; however, the role of CASK in the potential therapeutic value of osteosarcoma has not been reported.

**Methods:**

The Gene Expression Omnibus (GEO) database was used to analyze the expression levels of CASK in osteosarcoma patients. Cell experiments validated the role of CASK in osteosarcoma cells and revealed the potential mechanism of action of CASK from three aspects: immunity, pathogenic gene expression, and protein interaction.

**Results:**

The results showed that CASK was significantly upregulated in osteosarcoma samples. Inhibiting the expression of CASK can significantly promote cell apoptosis and inhibit the cycle progression of osteosarcoma cells. The high expression of CASK is mainly enriched in the immune system process, the JAK/STAT signaling pathway. There is a significant positive correlation between CASK and Macrophages_M2. Changes in CASK expression levels may activate the JAK/STAT signaling pathway by affecting the immunity of Macrophages_M2, thereby regulating apoptosis and cell cycle progression in osteosarcoma cells.

**Discussion:**

In conclusion, CASK may be a potential therapeutic target for osteosarcoma patients.

## 1 Introduction

Osteosarcoma is one of the most common malignant bone tumors and the second leading cause of cancer death in children and adolescents (1). At present, surgical resection and chemotherapy are the main treatment methods for osteosarcoma (2). Although the survival rate of osteosarcoma patients significantly improves after surgery combined with chemotherapy, the 5-year survival rate of patients is still not ideal (3, 4). Therefore, identifying new therapeutic targets is crucial for improving the treatment of osteosarcoma (5).

With the deepening of our research on osteosarcoma mechanisms, gene changes have become increasingly important in the pathology and immune mechanisms of osteosarcoma (6). Therefore, exploring the treatment of osteosarcoma at the genetic level is particularly important. The abnormal expression of Calcium/calmodulin-dependent serine protein kinase (CASK) plays an important role in the tumorigenesis of many cancers. CASK silencing inhibited cell proliferation, colony formation ability, and invasion and elicited apoptosis in pancreatic cancer cells (7). CASK is a promoter of prostate cancer progression and can enhance prostate cancer cell migration and invasion via kinase-dependent AKT activation (8). CASK protein was upregulated in pancreatic adenocarcinoma samples and cell lines and predicts poor outcomes in patients with pancreatic adenocarcinoma (9). However, the mechanism of CASK overexpression in osteosarcoma is still unknown. Based on these limited studies, the role of CASK in the mechanism of osteosarcoma is not yet clear; therefore, further research is needed to investigate the role of CASK.

This study first evaluated the differential expression changes of CASK in pan-cancer and then further evaluated the differential expression of CASK between the healthy control group and osteosarcoma patients. *In vitro* experiments were used to confirm the potential function of CASK in osteosarcoma cells. Finally, the potential mechanism of action of CASK was revealed from three aspects: pathways, immune, and protein interactions.

## 2 Materials and methods

### 2.1 Differential expression of CASK in pan-cancer

Gene Expression Profiling Interactive Analysis (GEPIA; http://gepia.cancer-pku.cn/index/html) was used to evaluate the RNA expression data of CASK. It can be used to evaluate the RNA that expression data of 8,587 normal The Cancer Genome Atlas (TCGA) samples and 9,736 tumor samples, as well as the Genotype-Tissue Expression (GTEx) database. When obtaining CASK’s gene expression profile, the ANOVA method was applied using the following thresholds: | log2FC | cutoff = 1, LogScale = log2 (TPM + 1), and q value cutoff = 0.01 (10).

### 2.2 Microarray data acquisition and processing in osteosarcoma

The gene expression microarray dataset used to evaluate the expression level of CASK includes the GSE42352 dataset and GSE33382 dataset. The GSE42352 dataset was obtained from the GPL10295 platform, including 15 normal samples and 103 osteosarcoma samples. The GSE33382 dataset was obtained from the GPL10295 platform, including three normal samples and 84 osteosarcoma samples (11). p ≤ 0.05 was considered statistically significant. The R 4.2.1 tool was used to evaluate gene expression levels by analyzing raw data from microarrays.

### 2.3 Construction of osteosarcoma cell model for CASK interference in U2OS cells

U2OS cells were sourced from the cell bank of The First Hospital of Jiaxing in Zhejiang Province. U2OS cells are cell lines of osteosarcoma cancer. Four siRNAs (U2OS + CASK-siRNA1, U2OS + CASK-siRNA2, U2OS + CASK-siRNA3, and U2OS + CASK-siRNA4) were constructed based on the CASK sequence. After transfection into U2OS cells, the interference effect of these three siRNAs on CASK was verified using qPCR. A random sequence was used as the control group (U2OS + siRNA-NC), and the cell model of CASK interference was constructed by selecting one of the four siRNAs with the best interference effect and a significant interference effect (12).

### 2.4 SiRNAs and CASK inhibitors were used to suppress the expression of CASK in osteosarcoma cells

Saos-2 cells were sourced from the cell bank of The First Hospital of Jiaxing in Zhejiang Province. To construct a CASK interference osteosarcoma cell model (Saos-2), two siRNAs (U2OS + CASK-siRNA1 and U2OS + CASK-siRNA4) were constructed based on the CASK sequence. After transfection into Saos-2 cells, the interference effect of these three siRNAs on CASK was verified using qPCR. A random sequence was used as the control group (U2OS + siRNA-NC), and the cell model of CASK interference was constructed by selecting one of the two siRNAs with the best interference effect and a significant interference effect. Western blotting (WB) was used to validate the cells of siRNA with the best interference effect and a significant interference effect. Meanwhile, to further validate the functional impact of inhibiting CASK expression on osteosarcoma cells (Saos-2), cells were treated with specific kinase inhibitors of CASK (NR162, 2 μM, 24 h) (8, 12, 13).

### 2.5 Western blotting

First, a 12% separation gel solution and a 5% concentrated gel were prepared. Protein samples were prepared by diluting in 5×loading buffer according to the ratio and boiling in water for 10 minutes. 20μl of the samples was added to each well and placed into an electrophoresis cell for 100 minutes (constant voltage of 90 V). Then, the transfer filter paper, gel, and Polyvinylidene Fluoride (PVDF) membrane were placed into the membrane transfer tank in sequence; the membrane transfer solution was added, the electrode was covered, and the voltage was adjusted to the maximum. Then, the membrane was transferred to a 1.5 mA/cm^2^ gel volume ice bath for 1.5 h. The membrane was blocked with Tris Buffered Saline with Tween 20 (TBST) containing 5% non-fat milk for 1 h at room temperature. The membrane was gently stirred with the required primary antibody at 4 °C overnight. After being washed with TBST three times for 10 minutes, the membrane was gently stirred at room temperature and incubated with the secondary antibody. After 2 h, the membrane was washed three times with TBST and soaked in Enhanced Chemiluminescence (ECL) luminescent solution for approximately 1 minute before being exposed to light in a dark room. Finally, film washing and scanning were performed (13).

### 2.6 Cell cycle experiments of osteosarcoma cells

The cells were collected after termination of digestion and centrifuged for 5 minutes (1,000 rpm). The supernatant was removed, resuspended, and washed twice with phosphate buffered saline (PBS). Precooled 80% ethanol at a volume of 700 μL was slowly added, fixed at 4°C for more than 4 h, centrifuged for 5 minutes (2,000 rpm), then rinsed twice with pre cooled PBS, added with 100 μL RNase (50 μg/mL), and placed in a water bath at 37 °C for 30 minutes. Propidium iodide (50 μg/mL) at a volume of 400 μL was added and stained at 4°C in the dark for 30 minutes before undergoing flow cytometry analysis (14).

### 2.7 Apoptosis experiments of osteosarcoma cells

The processed cells were collected and centrifuged for 5 minutes (1,500 rpm). The supernatant was removed and resuspended in PBS, and the cells were washed twice with PBS. A binding buffer at a volume of 500 μL was added to resuspend the cells. Then, 5 μL of Annexin V FITC was added and mixed well. Propidium iodide at a volume of 5 μL was added and allowed to react at room temperature in the dark for 5–15 minutes. Finally, flow cytometry was performed on the machine for detection (15).

### 2.8 Pathway analysis of CASK at the transcriptional level in osteosarcoma

To analyze the pathways of CASK in osteosarcoma, based on the GSE42352 dataset (correlation coefficient | R | > 0.3 and p < 0.05), the R software (psych package) was used to analyze Spearman’s correlation between CASK and other genes in all osteosarcoma patients, and then these screened genes were used for gene set enrichment analysis (GSEA) (ClusterProfiler package). GSEA used all patient samples from the dataset. False discovery rate (FDR) < 0.05 and normalized p-value < 0.05 were set as the threshold values (16).

### 2.9 Analysis of CASK- related protein interaction network

The GeneMANIA software (https://genemania.org/) was used to construct a Protein Protein Interaction (PPI) network centered on CASK. The PPI network included 20 CASK- related genes and CASK genes. Then, Kyoto Encyclopedia of Genes (KEGG) and Gene ontology (GO) function enrichment analyses were carried out for the gene that was centered on CASK, which was constructed using GeneMANIA (17).

### 2.10 Correlation analysis between CASK and immune cell infiltration

Based on the GSE42352 dataset (103 osteosarcoma samples), the CIBERSORT method and quantiseq method (Immunedeconv package) were used to analyze the infiltration level of immune cells. Subsequently, in order to evaluate the correlation between CASK and immune cell infiltration levels, Mantel’s test based on distance matrix (linker package in the R software) was used for analysis. Subsequently, Pearson’s correlation coefficient was used to calculate the correlation between the two distance matrices, and Mantel’s statistic size (Mantel’s r) and corresponding p-value (Mantel’s p) were obtained. The width of the line represents the range of Mantel’s r values, and the color of the line represents the level of p- values (Mantel’s p) (18–20). p-Value (Mantel’s p) < 0.01 was considered statistically significant.

## 3 Result

### 3.1 Expression of CASK in pan-cancer

**Figure 1:**
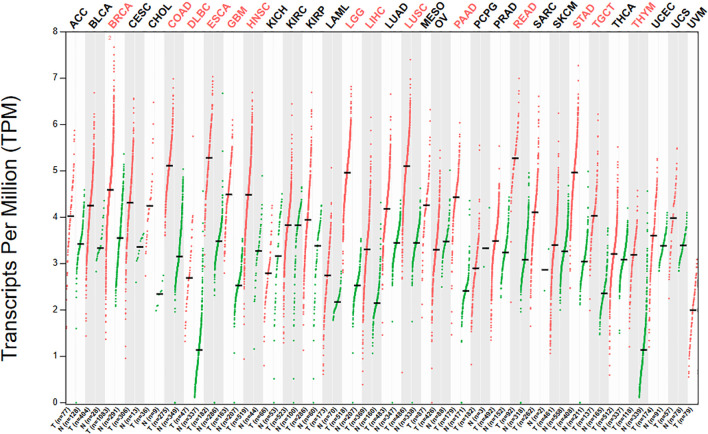
Expression of CASK in pan-cancer. We used GEPIA2 database, which integrated gene expression data from GTEx and TCGA to determine CASK’s expression level in pan-cancer. According to this result, compared with normal tissues, CASK’s expression in 14 types of cancer (BRCA, COAD, DLBC, ESCA, GBM, HNSC, LGG, LIHC, LUSC, PAAD, READ, STAD, TGCT, and THYM) was significantly upregulated. Red represents significant upregulation. We applied ANOVA method using the following thresholds: | log2F | Ccutoff = 1, LogScale = log2 (TPM + 1), and q value cutoff = 0.01. GTEx, Genotype-Tissue Expression; TCGA, The Cancer Genome Atlas; BRCA, breast invasive carcinoma; COAD, colon adenocarcinoma; DLBC, diffuse large B-cell lymphoma; ESCA, esophageal carcinoma; GBM, glioblastoma multiforme; HNSC, head and neck squamous cell carcinoma; LGG, low-grade glioma; LIHC, liver hepatocellular carcinoma; LUSC, lung squamous cell carcinoma; PAAD, pancreatic adenocarcinoma; READ, rectal adenocarcinoma; STAD, stomach adenocarcinoma; TGCTs, testicular germ cell tumors; THYM, thymoma.

According to this result, compared with normal tissues, CASK’s expression in 14 types of cancer [breast invasive carcinoma (BRCA), colon adenocarcinoma (COAD), diffuse large B-cell lymphoma (DLBC), esophageal carcinoma (ESCA), glioblastoma multiforme (GBM), head and neck squamous cell carcinoma (HNSC), low-grade glioma (LGG), liver hepatocellular carcinoma (LIHC), lung squamous cell carcinoma (LUSC), pancreatic adenocarcinoma (PAAD), rectal adenocarcinoma (READ), stomach adenocarcinoma (STAD), testicular germ cell tumors (TGCT), and thymoma (THYM)] was significantly upregulated (**Figure 1**). Red represents significant upregulation. The ANOVA method was applied using the following thresholds: | log2FC | cutoff = 1, LogScale = log2 (TPM + 1), and q value cutoff = 0.01. CASK may therefore be a potential oncogene. Upregulation of CASK may be one of the factors contributing to the occurrence of cancer.

### 3.2 CASK is a potential biomarker for osteosarcoma

**Figure 2:**
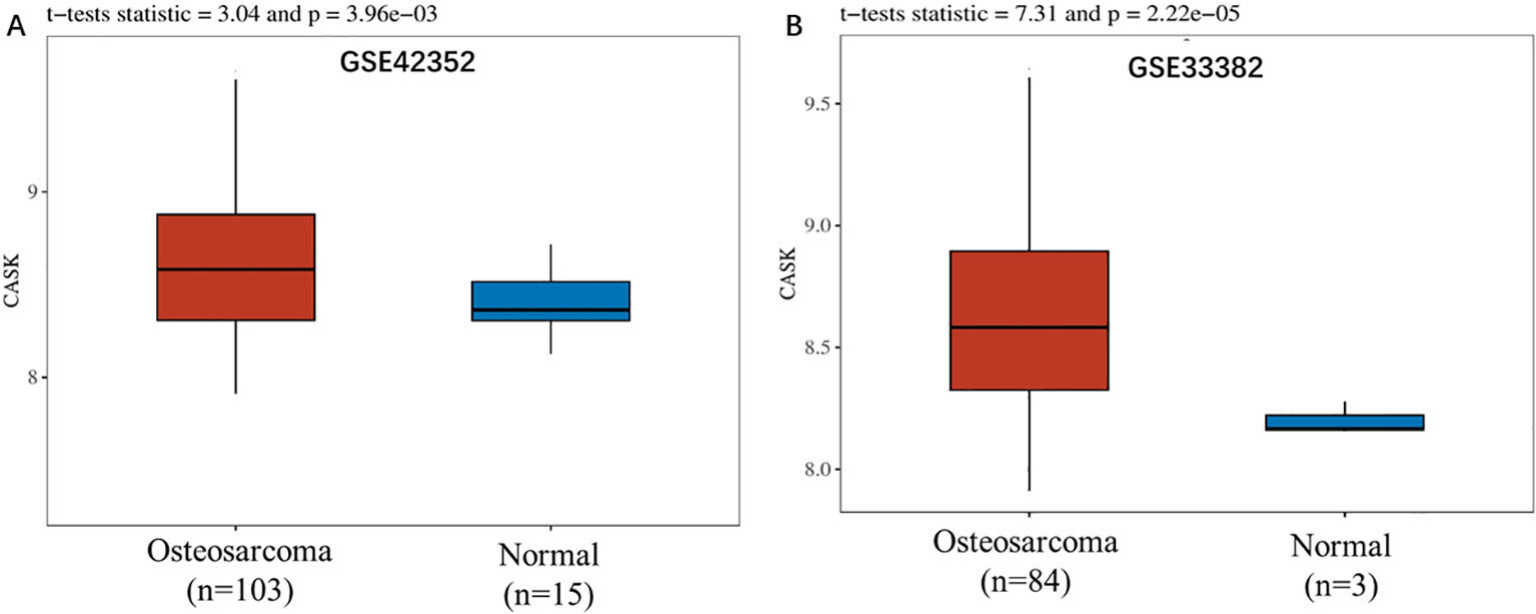
CASK is a potential biomarker for osteosarcoma. We analyzed CASK’s gene expression level in osteosarcoma using GSE42352. According to this result, CASK was upregulated in osteosarcoma samples compared to normal samples **(A)**. We therefore validated CASK’s differential expression in GSE33382 **(B)**. The statistical analysis results are displayed in a box plot, where red represents the osteosarcoma patient group (Osteosarcoma), and blue represents the control group (Normal). p ≤ 0.05 is considered to have a significant difference.

We analyzed CASK’s gene expression level in osteosarcoma using the GSE42352 dataset. According to this result, CASK was upregulated in cancer samples compared to normal samples (**Figure 2A**). We validated CASK’s differential expression in the GSE33382 dataset; CASK was upregulated in osteosarcoma samples compared to normal samples (**Figure 2B**). The statistical analysis results are displayed in a box plot, where red represents the osteosarcoma patient group (Osteosarcoma), and blue represents the control group (Normal). p ≤ 0.05 was considered statistically significant.

### 3.3 Construction of osteosarcoma cell model for CASK interference in U2OS cells

**Figure 3:**
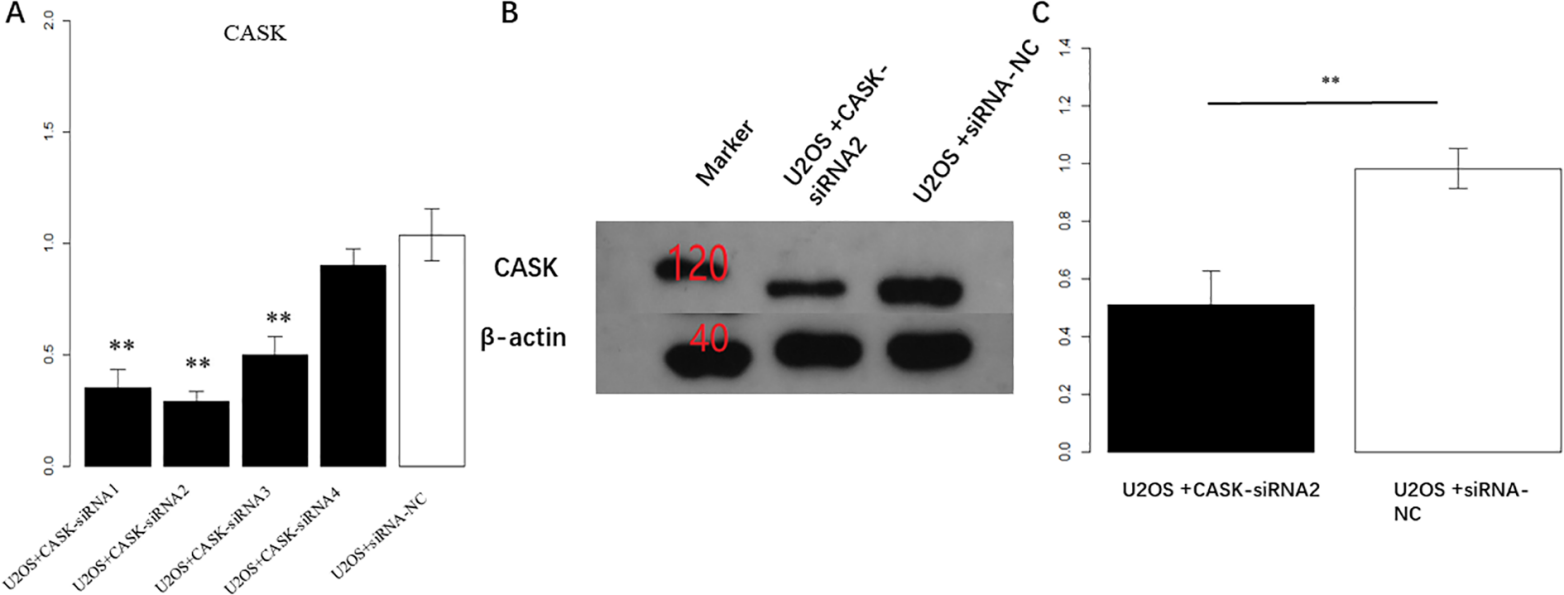
Construction of osteosarcoma cell model for CASK interference. The qPCR results showed that compared with the control group, the cells transfected with U2OS + CASK-siRNA2 had the most significant interference effect on CASK **(A)**. Western blotting experiments showed that compared to that in the control group, the expression level of CASK protein in the U2OS + CASK-siRNA2 group was significantly inhibited **(B, C)**. **p < 0.01.

The qPCR results showed that compared with the control group (U2OS + siRNA-NC), U2OS + CASK-siRNA1, U2OS + CASK-siRNA2, and U2OS + CASK-siRNA3 all showed significant interference differences (p < 0.01). Compared to U2OS + CASK-siRNA1 and U2OS + CASK-siRNA3, U2OS + CASK-siRNA2 had the smallest value and the most significant interference effect (**Figure 3A**). Therefore, the U2OS cells in the U2OS + CASK-siRNA2 group were used as a U2OS cell model. Meanwhile, Western blotting experiments showed that compared to the control group, the expression level of CASK protein in the U2OS + CASK-siRNA2 group was significantly inhibited (**Figures 3B, C**; **Supplementary Figure 1**). p < 0.05 was considered statistically significant. *p < 0.05, **p < 0.01, and ***p < 0.001.

### 3.4 Cell cycle experiments and apoptosis experiments in U2OS cells

**Figure 4:**
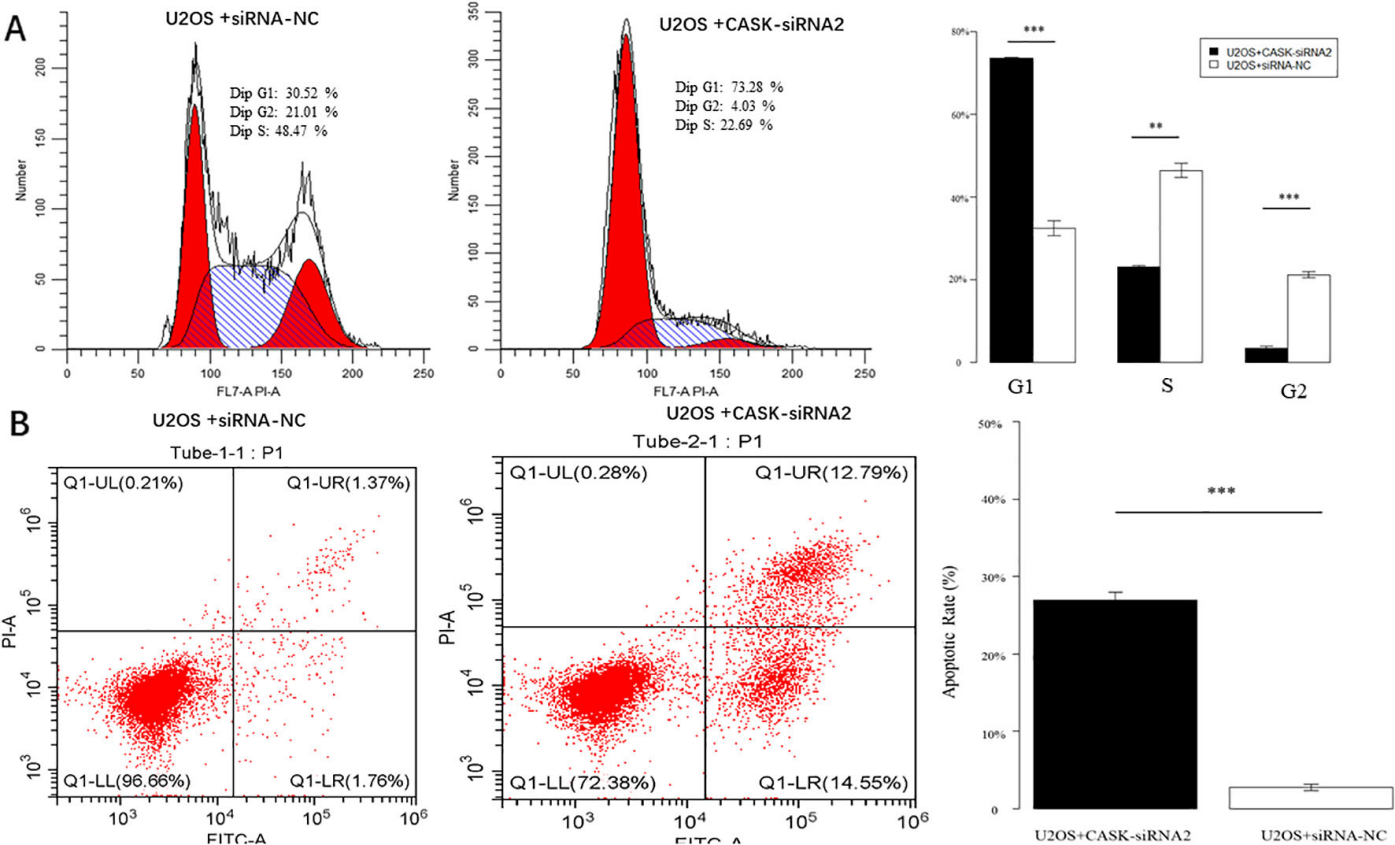
Cell cycle experiments and apoptosis experiments. The cell cycle results showed that compared with the U2OS + siRNA-NC group, the U2OS + CASK-siRNA2 group showed a significant increase in G1 phase cells, a significant decrease in S phase cells, and a significant decrease in G2 phase cells **(A)**. The results of cell apoptosis showed that compared with that of the U2OS + siRNA-NC group cells, the apoptosis rate of the U2OS + CASK-siRNA2 group cells was significantly increased **(B)**. **p < 0.01, and ***p < 0.001.

Cell experiments were used to investigate the potential function of CASK in osteosarcoma cells. Cell cycle analysis indicated that the U2OS + CASK-siRNA2 group exhibited inhibitory effects on U2OS cells in the G1 phase compared to the control group (U2OS + siRNA-NC) (**Figure 4A**). The results of cell apoptosis indicated a significant increase in the apoptosis rate of U2OS cells in the U2OS + CASK-siRNA2 group compared to the control group (U2OS + siRNA-NC) (**Figure 4B**). p < 0.05 was considered statistically significant. *p < 0.05, **p < 0.01, and ***p < 0.001.

### 3.5 Construction of osteosarcoma cell model for CASK interference in Saos-2

**Figure 5:**
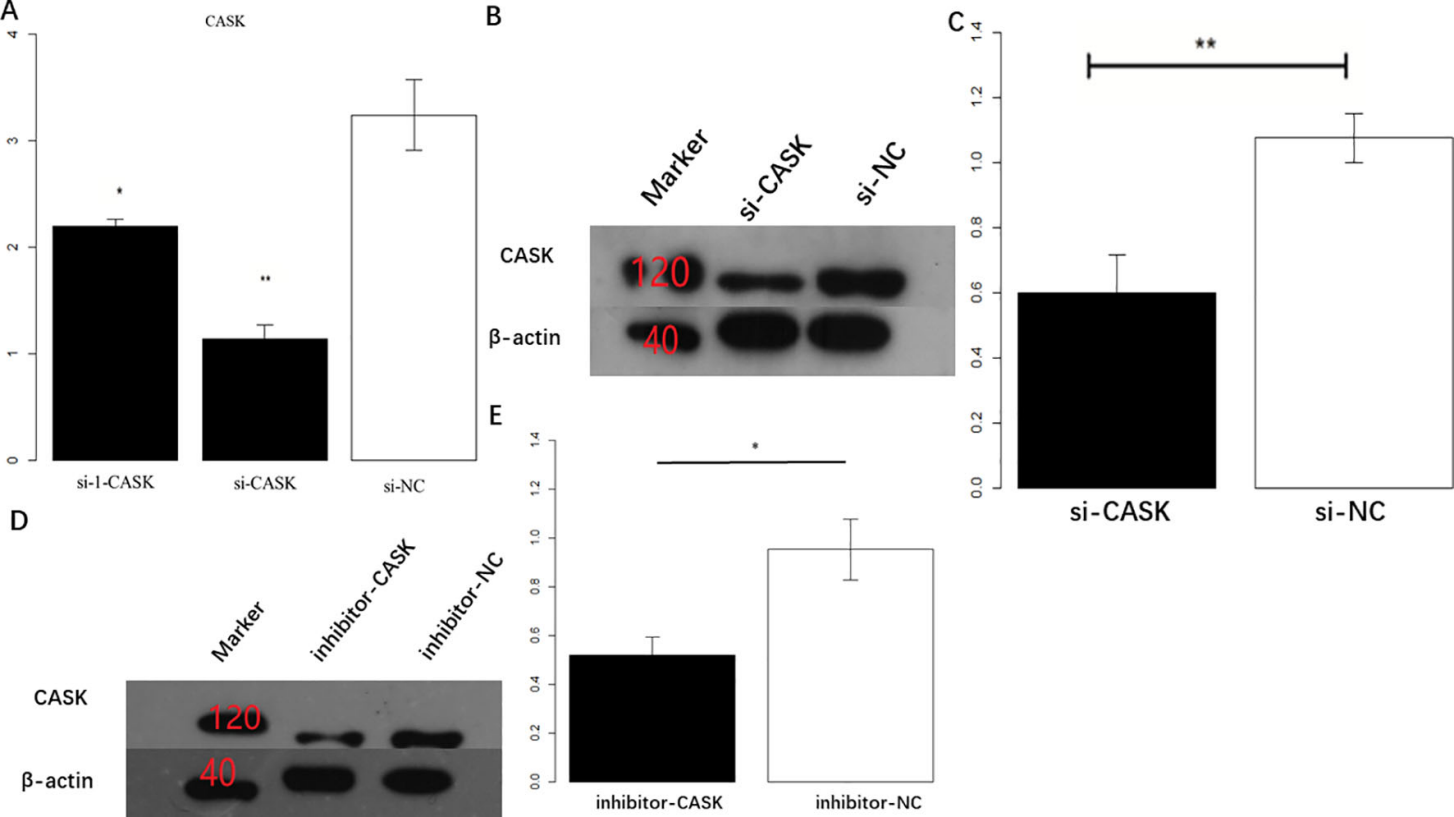
Construction of osteosarcoma cell model for CASK interference in Saos-2. The qPCR results showed that compared with the control group (si-NC), si-CASK has the smallest value and the most significant interference effect **(A)**. Western blotting experiments showed that compared to the control group, the expression level of CASK protein in the experimental group was significantly inhibited **(B, C)**. Western blotting experiments showed that compared to that in the control group (inhibitor-NC), the expression level of CASK protein in the inhibitor-CASK group was significantly inhibited **(D, E)**. p < 0.05 was considered statistically significant. *p < 0.05, **p < 0.01.

The qPCR results showed that compared with the control group (si-NC), si-CASK had the smallest value and the most significant interference effect (**Figure 5A**). Meanwhile, Western blotting experiments showed that compared to that in the control group, the expression level of CASK protein in the experimental group was significantly inhibited (**Figures 5B, C**, **Supplementary Figure 2**). Therefore, the Saos-2 cells in the si-CASK group were used as a Saos-2 cell model. Meanwhile, Western blotting experiments showed that compared to that in the control group (inhibitor-NC), the expression level of CASK protein in the inhibitor-CASK group was significantly inhibited (**Figures 5D, E**, **Supplementary Figure 3**). p < 0.05 was considered statistically significant. *p < 0.05, **p < 0.01, and ***p < 0.001.

### 3.6 Cell cycle experiments and apoptosis experiments in Saos-2 cells

**Figure 6:**
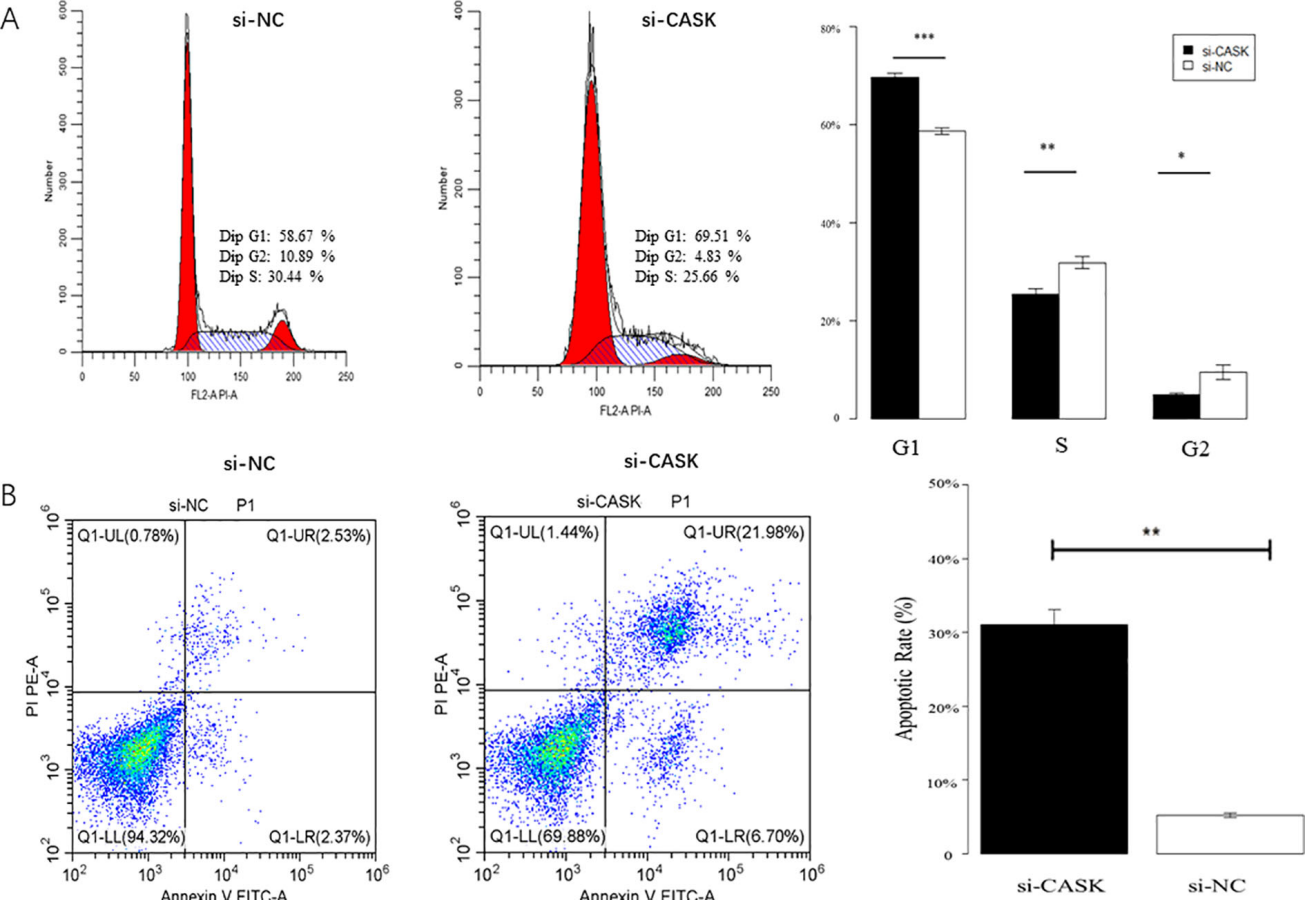
Cell cycle experiments and apoptosis experiments. Cell cycle analysis indicated that the si-CASK group exhibited inhibitory effects on Saos-2 cells in the G1 phase compared to the control group (si-NC) **(A)**. The results of cell apoptosis indicated a significant increase in the apoptosis rate of Saos-2 cells in the si-CASK group compared to the control group (si-NC) **(B)**. *P < 0.05, **P < 0.01, ***P < 0.001.

**Figure 7:**
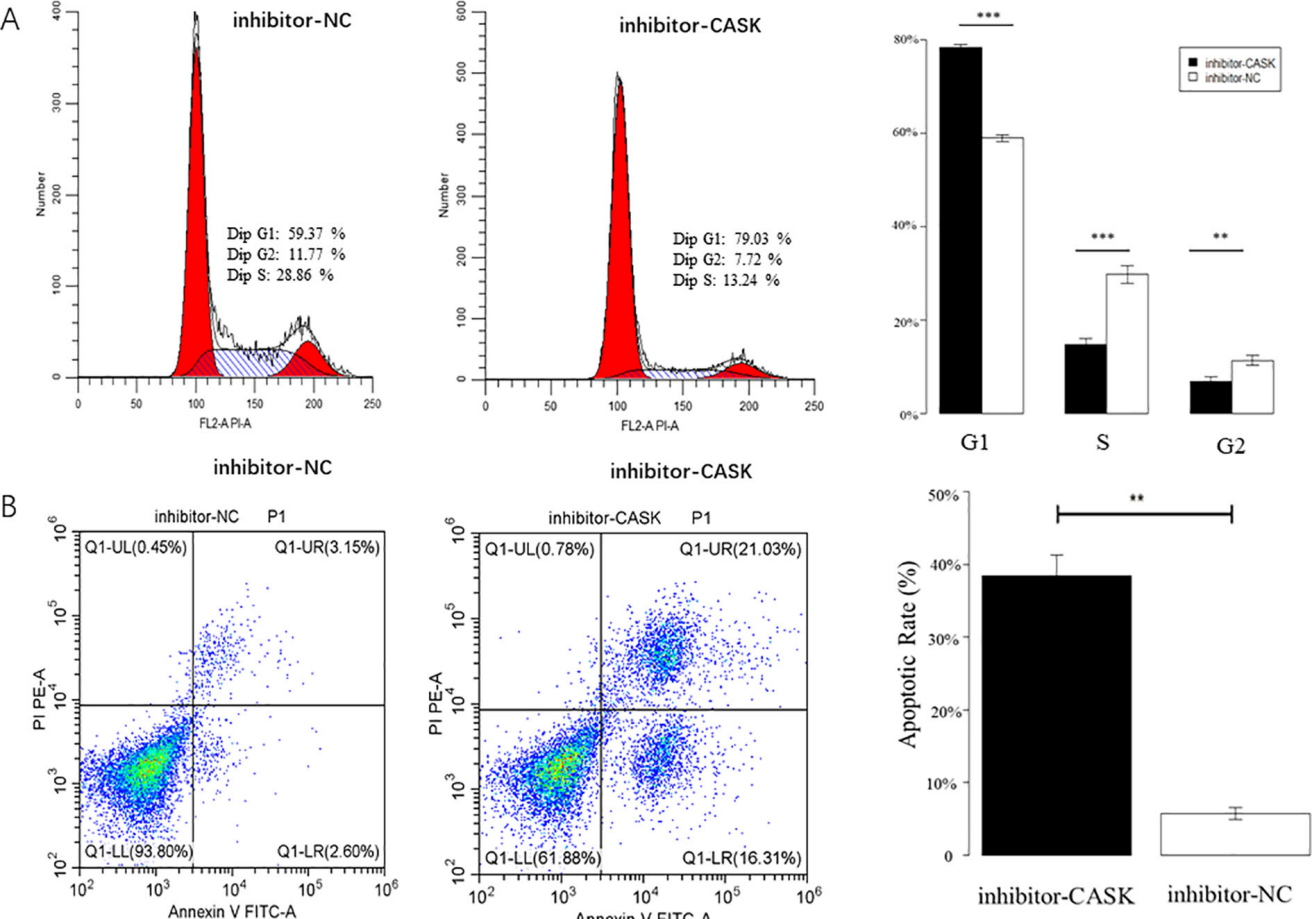
Cell cycle experiments and apoptosis experiments. The results of CASK inhibitor treatment on cells showed that compared with the control group, the use of the inhibitor significantly promoted cell apoptosis and inhibited cell cycle progression **(A, B)**. p < 0.05 was considered statistically significant. **p < 0.01, and ***p < 0.001.

Cell experiments were used to investigate the potential function of CASK in osteosarcoma cells. Cell cycle analysis indicated that the si-CASK group exhibited inhibitory effects on Saos-2 cells in the G1 phase compared to the control group (si-NC) (**Figure 6A**). The results of cell apoptosis indicated a significant increase in the apoptosis rate of Saos-2 cells in the si-CASK group compared to the control group (si-NC) (**Figure 6B**). Meanwhile, the results of CASK inhibitor treatment on cells showed that compared with the control group, the use of the inhibitor significantly promoted cell apoptosis and inhibited cell cycle progression (**Figures 7A, B**). p < 0.05 was considered statistically significant. *p < 0.05, **p < 0.01, and ***p < 0.001.

### 3.7 Pathway analysis of CASK at the transcriptional level and protein interaction network

**Figure 8:**
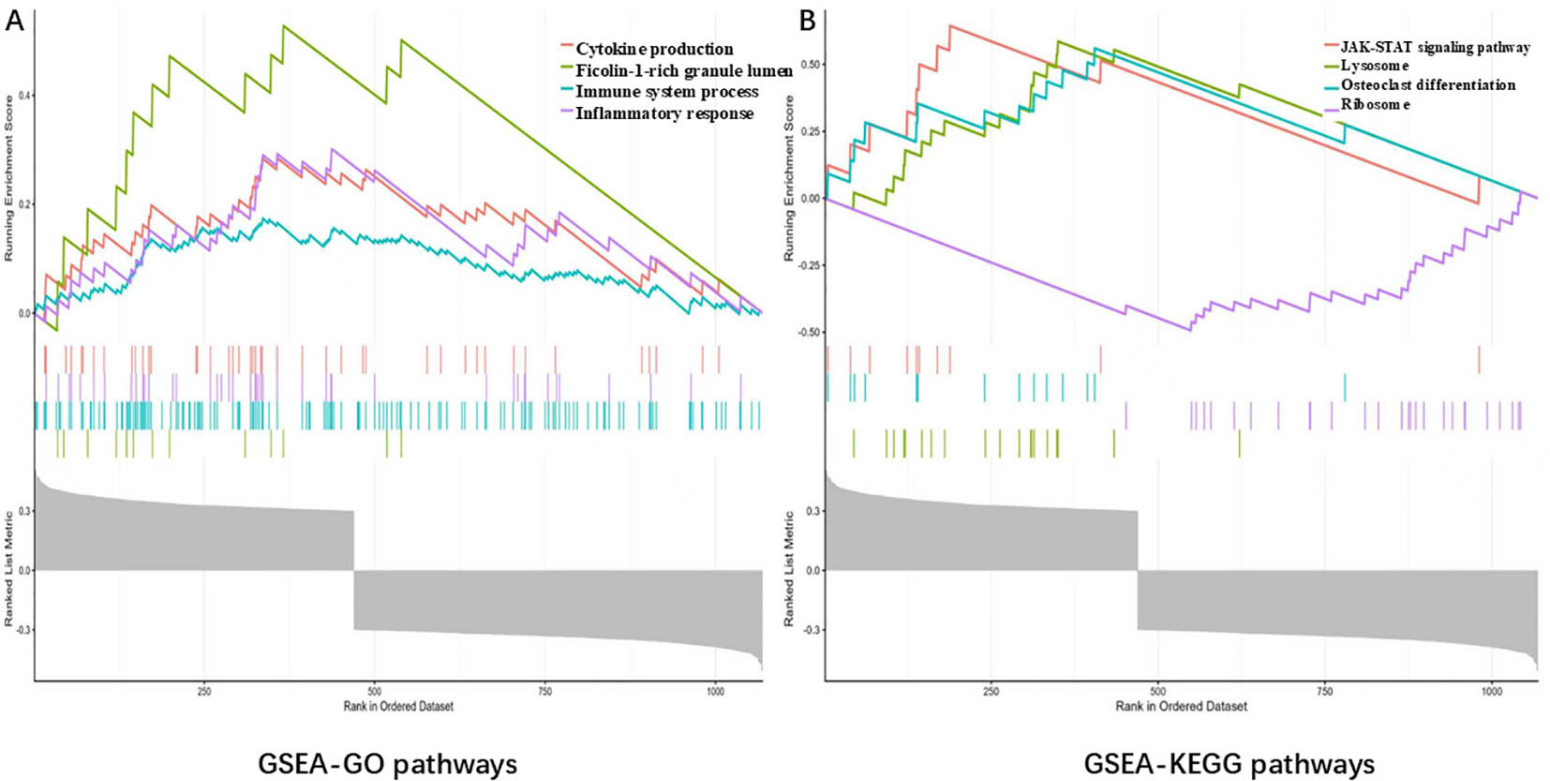
Pathway analysis of CASK at the transcriptional level in osteosarcoma. In the GSE42352 dataset, the high expression of CASK is mainly enriched in the ficolin-1-rich granule lumen, immune system process, inflammatory response, cytokine production **(A)**, and JAK/STAT signaling pathway **(B)**. False discovery rate (FDR) < 0.05 and normalized p- value < 0.05 were set as the threshold values.

**Figure 9:**
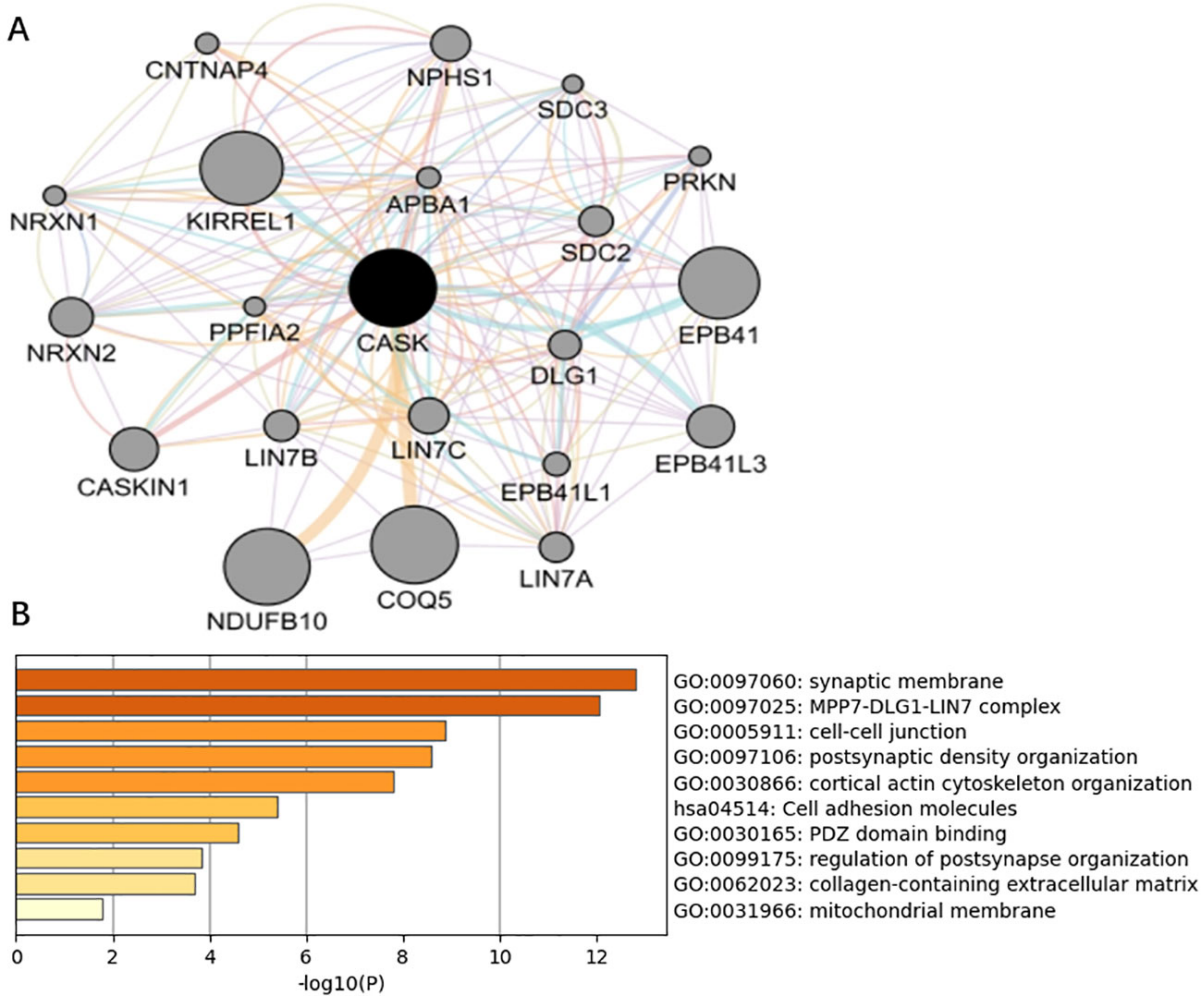
Analysis of CASK- related protein interaction network. GeneMANIA was used to build a PPI network of 21 genes (20 CASK- related genes and CASK) that were centered on CASK **(A)**. GO function enrichment analysis was carried out for these 21 genes **(B)**.

In the GSE42352 dataset (based on a sample of all 103 osteosarcoma samples), genes (484 positively associated genes and 620 negatively associated genes) significantly associated with CASK were screened for biological pathway analysis; the high expression of CASK is mainly enriched in the ficolin-1-rich granule lumen, immune system process, inflammatory response, cytokine production (**Figure 8A**), and JAK/STAT signaling pathway (**Figure 8B**). FDR < 0.05 and normalized p- value < 0.05 were set as the threshold values. A PPI network of 21 genes (20 CASK- related genes and CASK) was constructed using the GeneMANIA software (**Figure 9A**). A total of 21 genes were subsequently subjected to pathway enrichment analysis; the pathway analysis results showed that significantly enriched GO terms include synaptic membrane, MPP7–DLG1–LIN7 complex, and cell–cell junction (**Figure 9B**).

### 3.8 Correlation between CASK and immune cell infiltration

**Figure 10:**
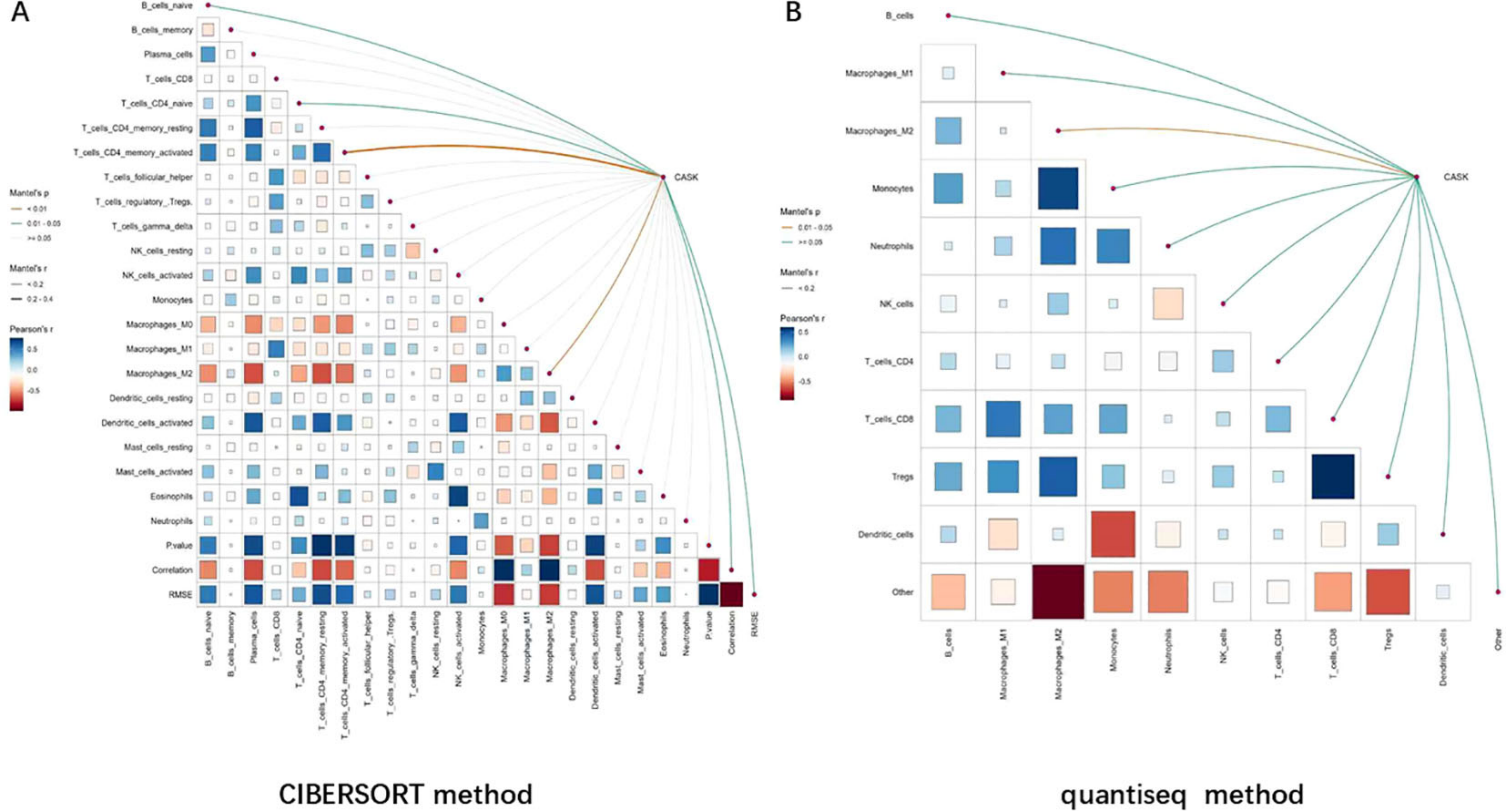
Correlation between CASK and immune cell infiltration. In the GSE42352 dataset, based on CIBERSORT method and quantiseq method, we found that CASK is significantly positively related to Macrophages_M2 **(A, B)**. p-value (Mantel’s p) < 0.01 was considered statistically significant.

We used the CIBERSORT method and the quantiseq method to determine whether the expression of CASK in osteosarcoma was related to immune cell infiltration. Based on the CIBERSORT method, we found that CASK is significantly positively related to Macrophages_M2 (Mantel’s p = 0.002) (**Figure 10A**). Based on the quantiseq method, we found that CASK is significantly positively related to Macrophages_M2 (Mantel’s p = 0.009) (**Figure 10B**). p-Value (Mantel’s p) < 0.01 was considered statistically significant.

## 4 Discussion

Currently, identifying new therapeutic targets in clinical practice is crucial for improving the treatment of osteosarcoma (21). This article aimed to explore the potential therapeutic value of CASK in osteosarcoma and provide a theoretical basis for screening new therapeutic targets for osteosarcoma in clinical practice. CASK was significantly upregulated in osteosarcoma patients (GSE42352 dataset), which was validated in an independent dataset (GSE33382 dataset). Subsequently, our osteosarcoma cell experiments confirmed that interfering with the expression of CASK can significantly promote apoptosis in osteosarcoma and inhibit its cell cycle progression. Therefore, CASK may be a potential therapeutic target in osteosarcoma.

To further explore the potential pathways and immune roles of CASK in osteosarcoma, we conducted pathway enrichment analysis and immune analysis. GSEA results showed that the high expression of CASK is mainly enriched in the immune system process, the JAK/STAT signaling pathway. Immune results showed that CASK is significantly positively related to Macrophages_M2. According to existing literature, macrophages within the tumor microenvironment are closely related to tumor progression, and Macrophages_M2 have been reported to play a pro-tumor role in the tumor microenvironment (22). Therefore, CASK is upregulated in osteosarcoma and may promote tumor progression by inducing Macrophages_M2 polarization in osteosarcoma. However, further experiments are needed to verify the specific mechanism.

The physiological significance of the JAK/STAT signaling pathway makes it an important target for treating various diseases, including autoimmune diseases, cancer, and viral infections (23). Meanwhile, the JAK/STAT signaling axis is closely related to tumor cell cycle, apoptosis, angiogenesis, inflammation, and other factors (24). Macrophages_M2 can promote tumor cell proliferation, survival, and drug resistance by activating signaling pathways such as JAK/STAT. For example, cytokines secreted by Macrophages_M2 (such as IL-6) bind to receptors and promote tumor cell proliferation and survival through the JAK/STAT signaling pathway (25). Therefore, changes in CASK expression levels may activate the JAK/STAT signaling pathway by affecting the immunity of Macrophages_M2, thereby regulating apoptosis and cell cycle progression in osteosarcoma cells. However, further experiments are needed to verify the specific mechanism.

## 5 Conclusion

This article aimed to explore the potential therapeutic value of CASK in osteosarcoma and provide a theoretical basis for screening new therapeutic targets for osteosarcoma in clinical practice. First, CASK was significantly upregulated in osteosarcoma patients (GSE42352 dataset), which was validated in an independent dataset (GSE33382 dataset). Inhibiting the expression of CASK can significantly promote cell apoptosis and inhibit the cycle progression of osteosarcoma cells. Second, GSEA results showed that the high expression of CASK is mainly enriched in the immune system process, the JAK/STAT signaling pathway. Immune results showed CASK is significantly positively related to Macrophages_M2. Changes in CASK expression levels may activate the JAK/STAT signaling pathway by affecting the immunity of Macrophages_M2, thereby regulating apoptosis and cell cycle progression in osteosarcoma cells. However, further experiments are needed to verify the specific mechanism. This study is mainly based on the Gene Expression Omnibus (GEO) database and may contain confounding factors. Therefore, the next step is to further validate the differential expression of CASK in osteosarcoma patients through prospective cohort studies and analyze the correlation between CASK and clinical characteristics of osteosarcoma patients. Second, *in vitro* cell experiments lack *in vivo* interactions and do not consider the regulation of the neuroendocrine system. Therefore, animal experiments will be conducted as the next step.

## Data Availability

Gene expression microarray data sets, including GSE42352 and GSE33382, are downloaded from the Gene Expression Omnibus (GEO) database (https://www.ncbi.nlm.nih.gov/geo/).
