# Supplementary material for: Identification and validation of the potential therapeutic value of CASK in osteosarcoma: a computational analysis and in vitro experiments
Source: Front Oncol. 2026 Mar 16;16:1693570. doi: 10.3389/fonc.2026.1693570 (PMC13033479; doi:10.3389/fonc.2026.1693570)
Supplement: Supplementary file 1 [file DataSheet1.docx]

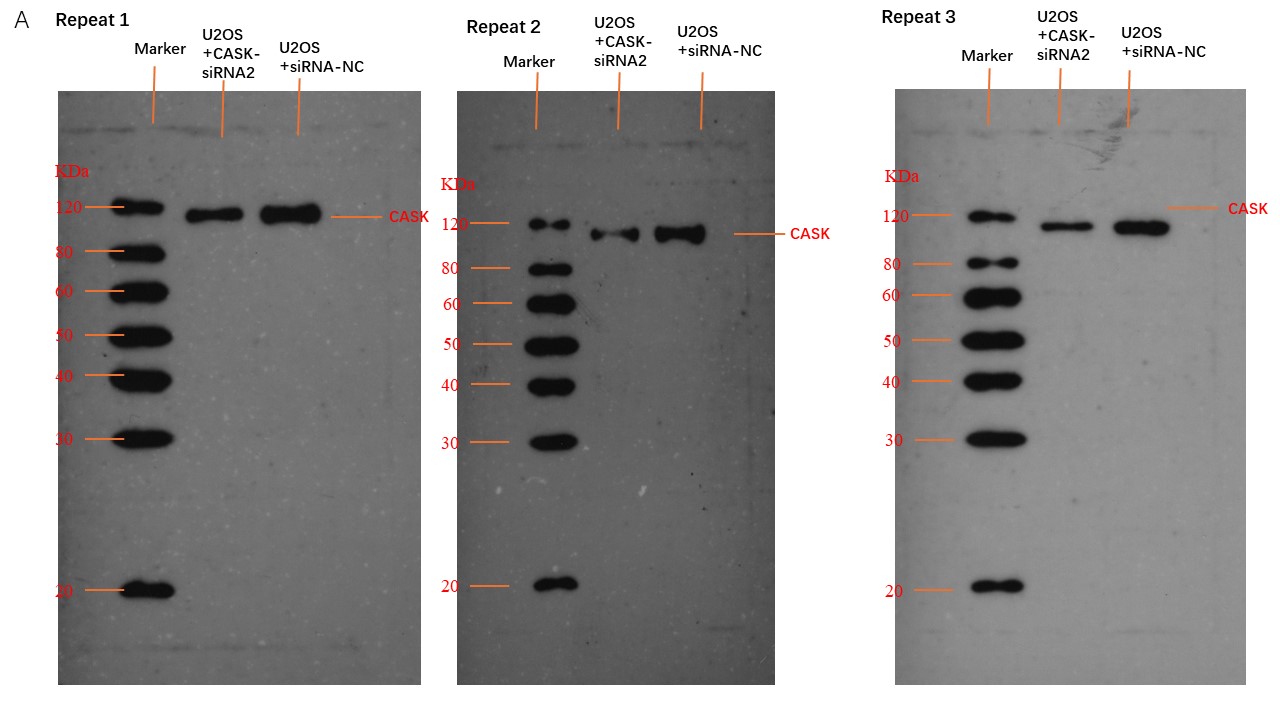


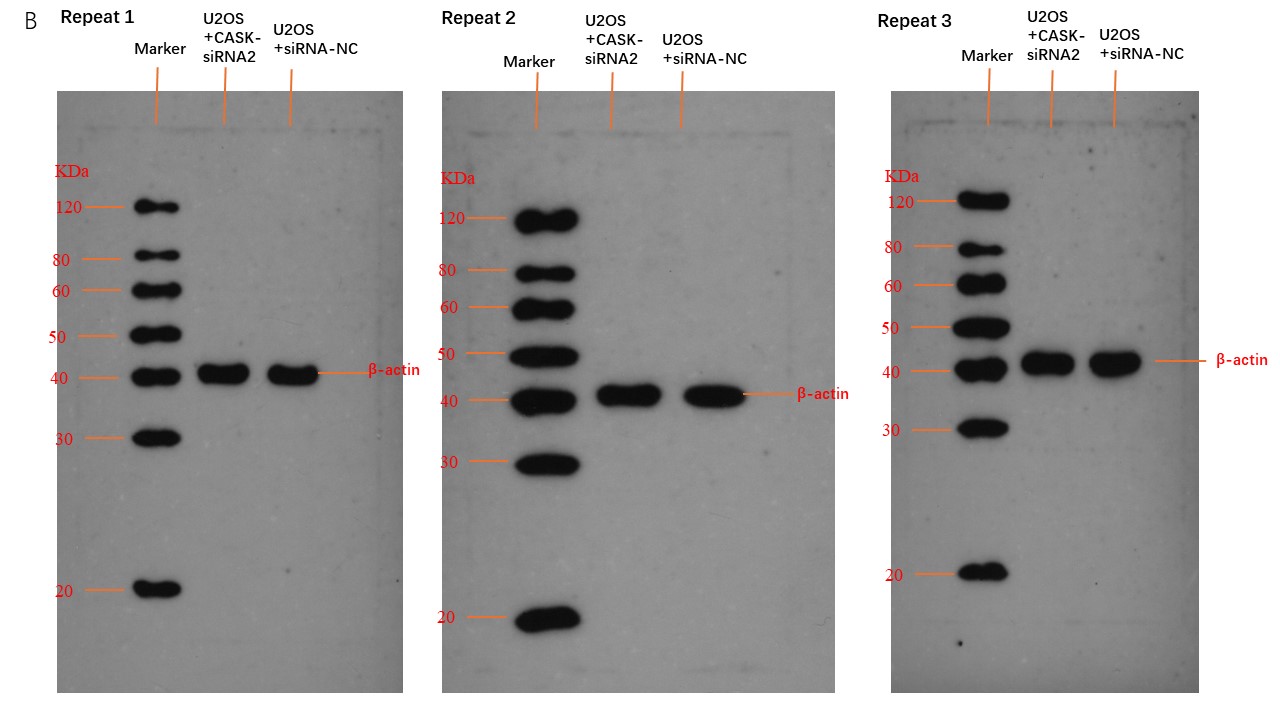


**Figure S1 Western blot experiment.**

The Western blot results showed that compared to the control group (U2OS+siRNA-NC), the U2OS+CASK-siRNA2 group was significantly down regulated (A-B).


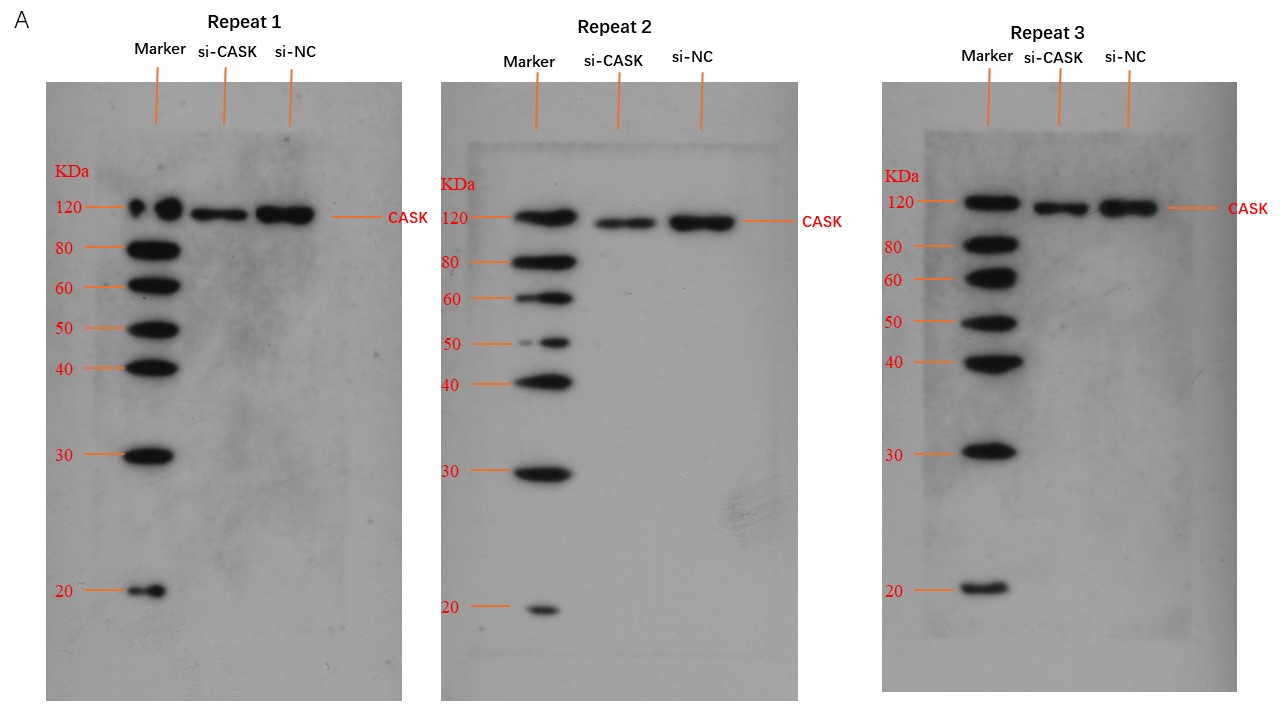


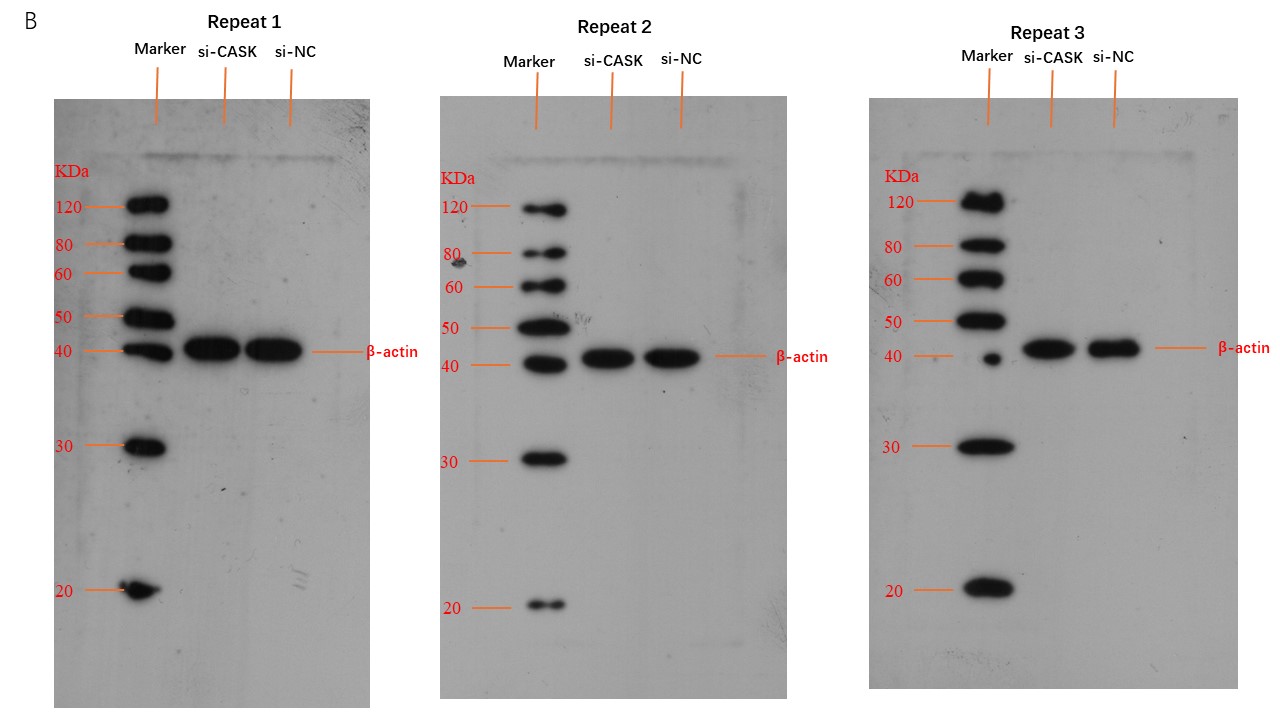


**Figure S2 Western blot experiment.**

The Western blot results showed that compared to the control group (si-NC), the si-CASK group was significantly down regulated (A-B).


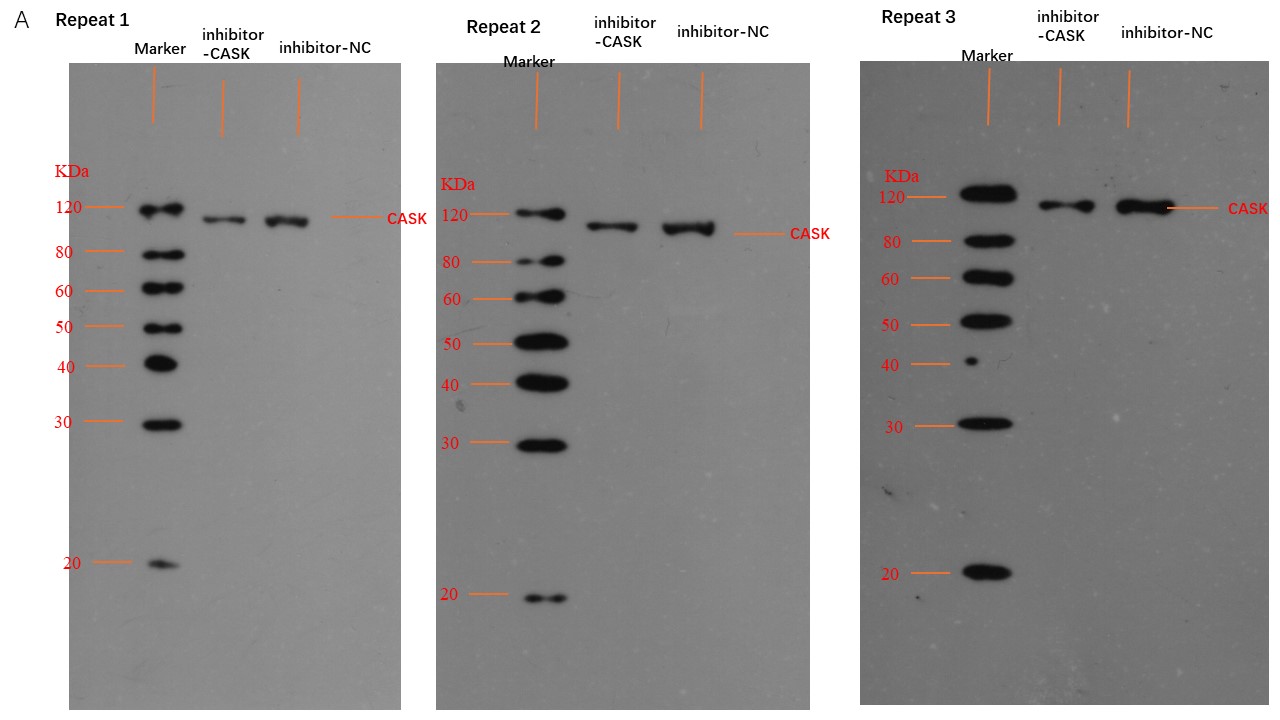


**
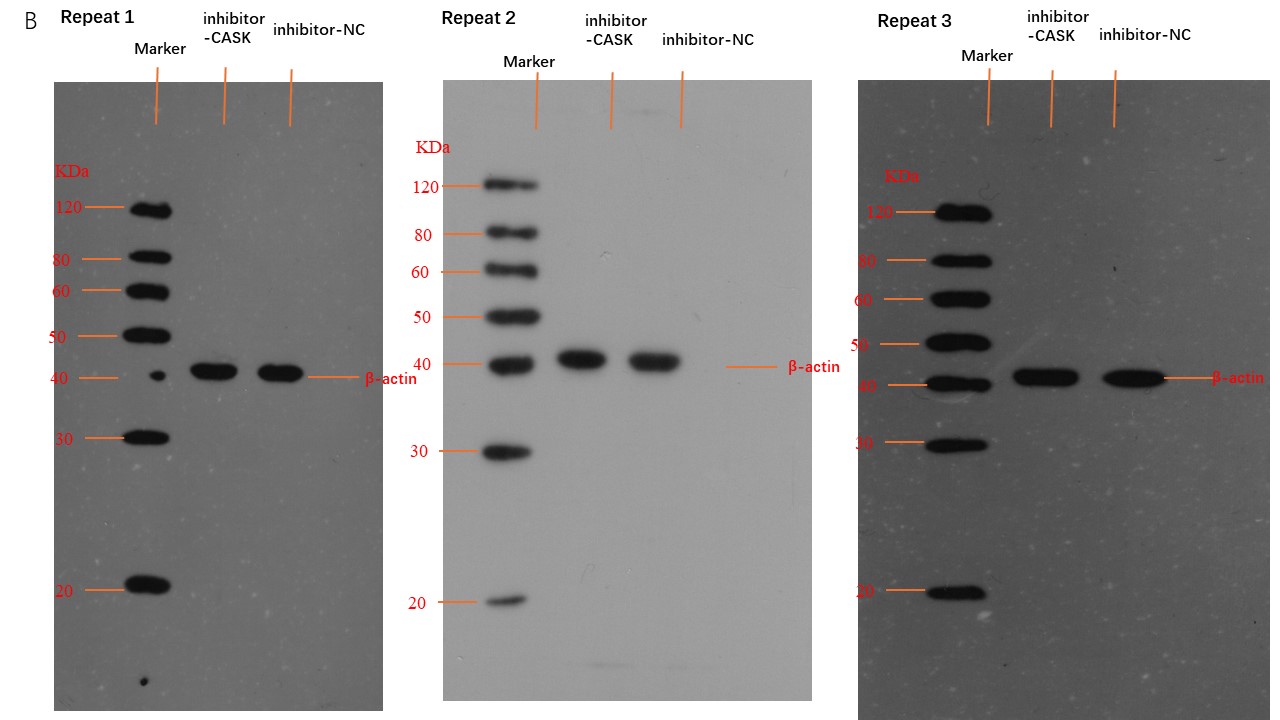
**

**Figure S3 Western blot experiment.**

The Western blot results showed that compared to the control group (inhibitor-NC), the inhibitor-CASK group was significantly down regulated (A-B).
